# Basic research in orthopedic surgery: Current trends and future directions

**DOI:** 10.4103/0019-5413.55969

**Published:** 2009

**Authors:** Chuanyong Lu, Jenni M. Buckley, Céline Colnot, Ralph Marcucio, Theodore Miclau

**Affiliations:** *Orthopaedic Trauma Institute, Department of Orthopaedic Surgery, University of California at San Francisco, San Francisco General Hospital, 2550 23rd St., San Francisco, CA 94110 USA*

Musculoskeletal problems continue to represent a growing source of death and disability world-wide, particularly with the growing burden of disease associated with an aging population and increase in the rates of road traffic accidents. To address the societal and economic burdens presented by musculoskeletal disorders, research in the normal biology of musculoskeletal tissues, the diseases and injuries associated with these tissues, and the underlying mechanisms of musculoskeletal tissue regeneration continue to gain importance. These investigations often require multidisciplinary approaches ranging from basic cellular and molecular biology, bioengineering, biomechanics, and clinical research. It is clear that collaboration between disciplines and centers with expertise in biology, mechanics, and clinical research is essential to continue to advance the field. The purpose of this review is to address issues that may be of interest to the development of new basic science research programs and initiatives, including a brief review of current and developing areas of orthopaedic research, and the resources required for the successful creation of new biology and mechanical research laboratories.

## DIRECTIONS IN ORTHOPEDIC RESEARCH

The musculoskeletal system involves a diverse organization of tissues exposed to a complex series of biological and mechanical stimuli. A thorough understanding of the normal biology of the musculoskeletal tissues, the behavior of these tissues associated with disease and injury, and the underlying mechanisms of musculoskeletal tissue regeneration is necessary to address the growing burden of disease. Research programs, both in developed and developing countries, must target those orthopedic conditions of greatest importance to their populations in order to diminish the societal and economic burdens caused by an inability to resume necessary physical function. The potential areas for investigation within the field of orthopedics continue to grow, particularly as the basic and applied body of scientific knowledge and technology develop. While these areas for basic research in orthopedics are too numerous to list, below are examples of some of the current and future directions in the field.

### Musculoskeletal injury and repair

Bone repair, whether it happens following a fracture or a bone graft, involves a well organized set of events that lead to reconstitution of the biological and mechanical integrity of bone. The regeneration process is initiated by an inflammatory response, which plays an important role in stimulating repair.^1^ Simultaneously, skeletal progenitor cells are recruited and begin differentiating into chondrocytes and osteoblasts that will deposit new cartilage and bone matrix necessary for bone bridging. The origins of these progenitor cells and the influence of the inflammatory response on their recruitment are not well understood. Following extracellular matrix deposition, cartilage is replaced by bone and new trabecular bone is converted to lamellar bone during the remodeling phase of repair.^2^–^4^

Numerous molecules and growth factors are keys to each step of the repair process and their functions are slowly being elucidated through analyses of various animal models of bone repair.^2^,^5^ These investigations will lead to a better understanding of the cellular and molecular bases of bone repair, better diagnosis of skeletal repair defects, and development of new strategies to accelerate healing. Surgeons have now the choice between various surgical techniques, improved implants and biological approaches to treat complex injuries. Current approaches use autografts, allografts, or bone morphogenetic proteins (BMPs). However, these approaches are not always successful and are costly, which necessitates the development of new therapies.

The muscles, tendons and ligaments along with blood vessels and nerves are closely associated with the bone. Musculoskeletal injuries may involve one or more of these tissues and the extent of injury is highly linked to the success of repair. For example, delayed union or non-union occurs in 5 to 10% of all fractures but is increased up to 46% in patients with extreme trauma and soft tissue damage.^6^ Therefore the role of numerous tissues must be taken in account in the majority of musculoskeletal diseases or injuries. Advances are being made in the basic biology of bone and individual soft tissues surrounding bone. The basic biology of muscle and muscle repair is well understood compared to other soft tissues. Muscle repair is composed of three phases including degeneration/ inflammation, regeneration and fibrosis. Many molecular markers and disease models are available. Muscle has been an ideal target to test new gene therapies and cell based therapies, however further advances are needed to treat devastating diseases such as Duchenne Muscular Dystrophy and to improve muscle repair. Vascular biology is also an area of intense investigation but more efforts need to be made to apply the data to the orthopedic field. The biology of tendons and ligaments is now being better understood with the identification of key molecular pathways involved in these tissues. Injury of tendons and/or ligament independent of bone can lead to complications and extended periods of recovery, which can also have debilitating effects. Like muscle and bone healing, tendon and ligament healing is initiated by an inflammatory response that may be modulated to stimulate repair. Little is know about the intrinsic capacities of tendon and ligament to heal and the cell sources that participate in repair.

### Cartilage biology

Regeneration of cartilage is a hot topic. Cartilage can be damaged by injury, inflammation, infection, and degeneration. Destruction of articular cartilage in rheumatoid arthritis and osteoarthritis involves inflammatory cytokines such as TNF-α, IL-1, or IL-6, which have been the target for current therapies.^7^–^11^ After injury, articular cartilage has a poor capacity to repair itself and tends to heal through the formation of fibrocartilage, which has inferior biomechanical characteristics to resist compression stress compared to normal articular hyaline cartilage. For decades scientists and surgeons have been exploring treatments that facilitate cartilage repair, including micro fractures and more recently cell-based tissue engineering using autologous chondrocytes and mesenchymal stem cells combined with scaffolds. Mesenchymal stem cells can be collected from many sources including bone marrow, adipose tissue, synovium, and muscles. Study on embryonic stem cells has also been initiated and this research direction could be very fruitful. Many cell factors may be exploited to improve these approaches, among which are TGF-β, IGF-1, FGF-2, and BMP-7.^12^ Nanotechonology may also improve the biomaterial properties of scaffolds. A better understanding of the cellular and molecular mechanisms of chondrocyte differentiation and phenotype maintenance may provide insights to develop novel techniques that can guide cultured chondrocytes and stem cells differentiation.

Regeneration of intervertebral discs (IVD) is equally, if not more challenging. A clinical study has shown that injection of disc chondrocytes reduces back pain and improves fluid contents of the treated disc.^13^ Experimental studies have demonstrated that injected mesenchymal stem cells can maintain viability and proliferate within the IVD.^14^ Although there are significant advances in the research of IVD regeneration, this area of research is still in its early stages. The intervertebral disc is composed of three tissues, the cartilage endplate, nucleus pulposus, and annulus fibrosus, and is more complex than articular cartilage. Successful regeneration of IVD may require the regeneration of all three tissues in one implant. The right scaffolds, cells, and techniques of implantation need to be determined and developed.^15^

### Orthopedic biomechanics

Orthopedic biomechanics is a specific sub-field of orthopedic research that involves the application of engineering principles to examine the mechanical behavior of the human musculoskeletal system. Topics of interest within orthopaedic biomechanics include mechanical testing of orthopaedic tissues and structures, medical implant design and testing, kinesiology (the study of human motion), and tissue engineering. A select list of currently popular research topics in Orthopedic Biomechanics for particular subspecialties is presented in Table 1.

**Table 1:** Examples of cutting-edge research topics in orthopedic biomechanical research

Orthopedic sub-specialty	
Spine	• Quantifying spinal kinematics in normal versus diseased cases
	• Understanding biomechanical contributions to adjacent segment disease
	• Comparison of motion preserving versus fusion technologies
Trauma and sports medicine	• Comparison of traditional versus less-invasive implants for bone fracture and soft tissue repair
	• Investigating the use of cements and adhesives to create more rigid fracture repair constructs that can support immediate weight bearing
Arthroplasty	• Optimization of bearing surfaces for total hip and knee prostheses
	• Geometric and bearing surface optimization for total disc and total ankle replacements
**Broad-based engineering topics**	
Computational Modeling	• Translating established continuum-based constitutive models for orthopedic tissues into finite element based algorithms
	• High-resolution finite element modeling of trabecular bone to study micro-scale damage accumulation
	• Multi-scale finite element modeling techniques to model bone-implant interfaces
	• Iterative finite element techniques to to integrate continuum-level effects of bone healing (Wolfe's Law) into traditional structural models of human bones and bone-implant systems
Wearable and implantable sensor systems	• Application of MEMS technology to traditional implant designs to measure loading *in-vivo*
Intra-operative navigation systems	• Comparison of the accuracy and mechanical integrity of bone-implant constructs performed using navigated percutaneous vs. traditional open techniques
Mechanical testing standards	• Development of appropriate testing standards for new implant designs in the spine, ankle, and upper extremities

MEMS = Microelectro mechanical system

In order to develop more effective surgical and non-surgical techniques for treating orthopedic diseases, orthopedic biomechanical research is performed to characterize the mechanical factors contributing to orthopaedic injury or resulting from underlying orthopaedic biological conditions. One example of this type of research is the investigation of the contribution of ligamentous structures to the stability of the knee^16^ or elbow^17^ and may apply to optimizing diagnostic tests for clinical instability or modifying joint replacement designs to better preserve surrounding soft tissues. Another active area of biomechanical research is the characterization of the mechanical threshold for micro- and macro-level bone failure. These studies are primarily focused on common anatomic sites of fragility fractures, such as the hip,^18^ distal radius^19^,^20^ and thoracolumbar spine^21^ and acute trauma of the skull,^22^ acetabulum^23^ and distal tibia and fibula. Lastly, there is a large body of research focused on characterizing differences between normal versus diseased orthopedic tissues at the tissue-level, such as elastic compressive behavior of the annulus fibrosus as a function of intervertebral disc degeneration.^24^,^25^ The potential applications of this work include specifying design parameters for tissue-engineered implants, quantifying the effects of different drug therapies on tissue-level mechanical behavior, and providing accurate material property information for computational models.

The application of new technology to the prevention and treatment of orthopaedic disease is another ongoing area of research in orthopedic biomechanics. Advancements in medical imaging, such as rapid-scanning MRI and low radiation dosage CT scans, have allowed for the development of patient-specific volumetric models of specific anatomy that can be used for pre-operative planning or injury prevention. For example, there have been clinical^26^ and cadaver-based biomechanical studies demonstrating the benefits of pre-operative planning using CT-based 3-D image processing for periacetabular osteotomy surgery (PAO) software to reduce operative time and improve surgical outcomes. Additionally, biomechanical studies have been instrumental in integrating recent advancements in materials science into orthopaedic implant design. For example, shape-memory alloys are now being used increasingly in minimally-invasive spinal surgeries, and cadaver-based biomechanical studies have been instrumental in demonstrating the safety and efficacy of these new implants.

Finally, a substantial portion of orthopedic biomechanical research is focused on the evaluation of existing orthopaedic techniques. These studies frequently involve head-to-head comparisons of the ex-situ or in-situ mechanical performances of currently used orthopedic implants or surgical techniques. These types of studies determine which procedure is most mechanically competent, and the data can be interpreted in the context of any existing clinical information regarding relative rates of patient morbidity and mortality, the amount of surgical skill needed to perform the procedure, and cost and availability of any technology necessary to perform the procedure. Examples of this type of research include overload of flexor tendons repaired with different stitching techniques^27^ and the effect of bone cement to augment laterally plated tibial plateau constructs.

## DEVELOPMENT OF AN ORTHOPEDIC RESEARCH PROGRAM

The initiation of research programs requires complex decision-making as directional, logistical, financial, and other considerations must be evaluated. The greatest barriers to the development of new basic research facilities include available technical expertise, space, and finances. This section reviews the basic infrastructure and equipment needs for the development of orthopedic molecular biology and biomechanical research laboratories, as well as some of the financial considerations required to develop these facilities.

### Infrastructure and equipments: Molecular biology laboratory

The infrastructure required to run an Orthopaedic Surgery Research laboratory is similar to any other biological laboratory. Fume hoods are required to vent noxious and dangerous chemicals. An animal housing facility is necessary if work is performed on any number of model organisms. If work is to be performed on established or primary cell lines, then a separate cell culture room should be considered. By isolating cell culture facilities, reduced foot traffic in and around the incubators and hoods will aid in keeping cultures free of bacteria and mold. Another part of the laboratory should be set aside for processing, sectioning, and staining of histological specimens. This area should be located in a “dust-free” area away from drafts that will create difficulty handling ribbons of sections. Work with radioactive materials can be made safer by defining and restricting use of these materials to dedicated areas of the laboratory. Similarly a dedicated imaging suite that contains all the microscopes that will be used for documentation and analysis of data will allow undisturbed specimen viewing, will allow the room to be darkened for specialized imaging such as epifluorescence, and will reduce the amount of dust that accumulates on working parts of the microscope.

Equipment for an Orthopedic Surgery laboratory performing molecular and cellular biology experiments includes microtomes, thermal cyclers, bacterial shaking incubators and incubator ovens, electrophoresis equipment for assessing DNA, RNA, and proteins, table top microfuge, centrifuge, safety cabinets to store flammable liquids. If work is primarily focused on in vitro analyses, then cell culture incubator(s), laminar flow hoods, and at least one inexpensive inverted phase contrast microscope for visualizing cells are required. More specialized equipment can be used as the necessity of the laboratory dictates. For example, quantitative reverse transcriptase PCR (qPCR) can be used to assess gene expression patterns in cells and tissues.

### Infrastructure and equipment: Orthopedic biomechanics laboratory

Research programs of Orthopedic Biomechanics generally have either experimental or computational focus, with a select group of more established laboratories undertaking both investigative approaches. The computational approach is generally preferred by new research groups in the US and Europe with limited financial resources. It can be established with minimal investment in infrastructure [Table 2] and may be staffed effectively by mechanical or computer engineers with basic familiarity with 3-D software and finite element techniques. As projects are fundamentally computer-based, collaborations may be established internationally with communication largely through video conferencing, secure file transfer protocols, and other forms of electronic communication.

**Table 2:** Recommended lab design for developing orthopedic biomechanics programs (Designs for both computationally-focused and experimentally-focused programs are provided)

Computational	
Laboratory space	• A desk in an office
Specialized equipment	• 1 computer workstation (dual-processor plus graphics card recommended)
	• 1 software license for a finite element program with pre and post-processing capabilities (Abaqus recommended)
Staffing	• 1 professional engineer or advanced engineering student (MS or PhD-level recommended) from a mechanical or computer engineering background
Experimental	
Laboratory space	• Minimum of 400 assignable square feet
	• Certified for handling of human and animal tissue
	• Temperature-controlled and well ventilated
Specialized equipment	• 1 axial or axial-torsional mechanical testing system. Instron or MTS recommended (USD$150K), although less expensive models are available (TestResources, USD$10K-$20K)
	• High (20 kN) and low (100 N) capacity uniaxial load cells (1 each). Multi-axial load cells necessary for off-axis testing protocols (USD$6K, AMTI recommended)
	• Household refrigerators and freezers for specimen storage
Staff	• Minimum of one full-time professional engineer and one research assistant (may be a student) to conduct experiments
	• 6-9 months of on-the-job training is necessary for staff to become proficient in running experimental protocols
Other considerations	• A source of cadaveric tissue or animal tissue is needed for *in vitro* experimental protocols
	• Partnerships with orthopaedic device companies, are frequently necessary in order to test orthopaedic implants
	• Access to standard clinical radiographic equipment is necessary. Particularly DEXA, planar x-ray, and occasionally CT

Experimentally-focused orthopedic biomechanics research programs are substantially more challenging to develop, as they involve substantial up-front investment in laboratory infrastructure, have high operational costs, and require close clinical collaboration and experienced technical staff. Well-established, experimentally-based biomechanics programs in the US and Europe typically: 1) own $500K-$1M (United States Dollars, USD) of custom laboratory equipment; 2) occupy temperature-controlled, 1000+ assignable square foot laboratory spaces with Biosafety-level 2 certification to handle human cadaveric tissue; 3) have two to four full-time staff. It is possible for new programs to successfully participate in the international research community with a scaled-down version [Table 2] of the aforementioned “established” experimental laboratory design; however, these programs must carefully select research projects to not over-tax their in-house resources.

## RESEARCH COLLABORATIONS AND OPPORTUNITIES

Numerous countries around the world have highly developed infrastructures for the support of basic research. While there are often opportunities for young scientists to obtain research training in these countries, including graduate and post-graduate instruction, perhaps the greatest long-term opportunities exist through the creation of productive collaborations. The first step to assuring a successful collaboration is to define a specific question and identify particular needs, which can be adapted to the local environment based on the available techniques, expertise, and models. The perceived needs should be well-defined before seeking outside expertise, and will facilitate the identification of appropriate collaborators. Potential collaborators may be identified through research or other professional societies, publications, or scientific meetings. In the US, for example, such collaborations could be identified through organizations such as the Orthopedic Research Society or the Society of Mechanical Engineers Bioengineering Division and journals such as the Journal of Orthopedic Research or Journal of Biomechanics, where the major orthopedic research centers in the United States are generally represented. New techniques or models may be learned via sending laboratory members to the collaborating laboratory. More advanced collaboration may be based on sharing research efforts on a project and might involve the acquisition of funding from one or all of the collaborating centers.

## CONCLUSIONS

The field of orthopedic research will continue to grow in order to address the increasing global burden of musculoskeletal injury and disease. New basic scientific discoveries in biological and mechanical research will continue to advance rapidly, and present opportunities to bring these new discoveries to the clinic. The complex nature of the musculoskeletal system requires multi-disciplinary collaborations between investigators that possess a wide diversity of expertise. Although the development of research laboratories and opportunities require extensive planning and resource development, ultimately basic discoveries have the potential to develop into translational projects that can impact patient care. Several such discoveries have already developed into large-scale multi-national clinical trials, which are the end-goal for basic science research.

## References

[1] Einhorn TA, Majeska RJ, Rush EB, Levine PM, Horowitz MC (1995). The expression of cytokine activity by fracture callus. J Bone Miner Res.

[2] Einhorn TA (1998). The cell and molecular biology of fracture healing. Clin Orthop Relat Res.

[3] Miclau T, Bozic K, Tay BK, Kim HG, Colnot C, Puttlitz C, Einhorn T, O'Keefe RJ, Buckwalter JA (2007). Bone injury, regeneration and repair. Orthopaedic basic science.

[4] Colnot C, Thompson Z, Miclau T, Werb Z, Helms JA (2003). Altered fracture repair in the absence of MMP9. Development.

[5] Einhorn TA (1999). Clinically applied models of bone regeneration in tissue engineering research. Clin Orthop Relat Res.

[6] Sen MK, Miclau T (2007). Autologous iliac crest bone graft: Should it still be the gold standard for treating nonunions?. Injury.

[7] Boissier MC, Assier E, Biton J, Denys A, Falgarone G, Bessis N (2009). Regulatory T cells (Treg) in rheumatoid arthritis. Joint Bone Spine.

[8] Brennan FM, McInnes IB (2008). Evidence that cytokines play a role in rheumatoid arthritis. J Clin Invest.

[9] Oldfield V, Dhillon S, Plosker GL (2009). Tocilizumab: A review of its use in the management of rheumatoid arthritis. Drugs.

[10] Smolen JS, Kay J, Doyle MK, Landewé R, Matteson EL, Wollenhaupt J (2009). Golimumab in patients with active rheumatoid arthritis after treatment with tumour necrosis factor alpha inhibitors (GO-AFTER study): A multicentre, randomised, double-blind, placebo-controlled, phase III trial. Lancet.

[11] Goldring SR, Goldring MB (2004). The role of cytokines in cartilage matrix degeneration in osteoarthritis. Clin Orthop Relat Res.

[12] Getgood A, Brooks R, Fortier L, Rushton N (2009). Articular cartilage tissue engineering: Today's research, tomorrow's practice?. J Bone Joint Surg Br.

[13] Meisel HJ, Ganey T, Hutton WC, Libera J, Minkus Y, Alasevic O (2006). Clinical experience in cell-based therapeutics: intervention and outcome. Eur Spine J.

[14] Crevensten G, Walsh AJ, Ananthakrishnan D, Page P, Wahba GM, Lotz JC (2004). Intervertebral disc cell therapy for regeneration: Mesenchymal stem cell implantation in rat intervertebral discs. Ann Biomed Eng.

[15] Kandel R, Roberts S, Urban JP (2008). Tissue engineering and the intervertebral disc: The challenges. Eur Spine J.

[16] Woo SL, Fisher MB (2009). Evaluation of knee stability with use of a robotic system. J Bone Joint Surg Am.

[17] Regan W, Lapner PC (2006). Prospective evaluation of two diagnostic apprehension signs for posterolateral instability of the elbow. J Shoulder Elbow Surg.

[18] Keyak JH, Rossi SA, Jones KA, Skinner HB (1998). Prediction of femoral fracture load using automated finite element modeling. J Biomech.

[19] Njeh CF, Wu C, Fan B, Hans D, Fuerst T, He Y (2000). Estimation of wrist fracture load using phalangeal speed of sound: an in vitro study. Ultrasound Med Biol.

[20] Wu C, Hans D, He Y, Fan B, Njeh CF, Augat P (2000). Prediction of bone strength of distal forearm using radius bone mineral density and phalangeal speed of sound. Bone.

[21] Buckley JM, Cheng L, Loo K, Slyfield C, Xu Z (2007). Quantitative computed tomography-based predictions of vertebral strength in anterior bending. Spine.

[22] Yoganandan N, Pintar FA (2004). Biomechanics of temporo-parietal skull fracture. Clin Biomech.

[23] Olson SA, Bay BK, Hamel A (1997). Biomechanics of the hip joint and the effects of fracture of the acetabulum. Clin Orthop Relat Res.

[24] Elliott DM, Setton LA (2001). Anisotropic and inhomogeneous tensile behavior of the human anulus fibrosus: Experimental measurement and material model predictions. J Biomech Eng.

[25] Lotz JC, Hsieh AH, Walsh AL, Palmer EI, Chin JR (2002). Mechanobiology of the intervertebral disc. Biochem Soc Trans.

[26] Hsieh PH, Chang YH, Shih CH (2006). Image-guided periacetabular osteotomy: Computer-assisted navigation compared with the conventional technique: A randomized study of 36 patients followed for 2 years. Acta Orthop.

[27] Rees L, Matthews A, Masouros SD, Bull AM, Haywood R (2009). Comparison of 1- and 2-knot, 4-strand, double-modified kessler tendon repairs in a porcine model. J Hand Surg Am.

